# Thermostability modification of β-mannanase from *Aspergillus niger via* flexibility modification engineering

**DOI:** 10.3389/fmicb.2023.1119232

**Published:** 2023-02-20

**Authors:** Shundong Tan, Xiumei Tao, Pu Zheng, Pengcheng Chen, Xiaowei Yu, Ning Li, Tiecheng Gao, Dan Wu

**Affiliations:** ^1^Key Laboratory of Industrial Biotechnology, Ministry of Education, School of Biotechnology, Jiangnan University, Wuxi, China; ^2^State Key Laboratory of Food Science and Technology, School of Food Science and Technology, Jiangnan University, Wuxi, China; ^3^Guangzhou Puratos Food Co., Ltd., Guangzhou, China

**Keywords:** β-mannanase, *Pichia pastoris*, molecular dynamics simulation (MD), thermostability, rational design

## Abstract

**Introduction:**

β-Mannanases can hydrolyze mannans, which are widely available in nature. However, the optimum temperature of most β-mannanases is too low to be directly utilized in industry.

**Methods:**

To further improve the thermostability of Anman (mannanase from *Aspergillus niger* CBS513.88), B-factor and Gibbs unfolding free energy change were used to modify the flexible of Anman, and then combined with multiple sequence alignment and consensus mutation to generate an excellent mutant. At last, we analyzed the intermolecular forces between Anman and the mutant by molecular dynamics simulation.

**Results:**

The thermostability of combined mutant mut5 (E15C/S65P/A84P/A195P/T298P) was increased by 70% than the wild-type Amman at 70°C, and the melting temperature (Tm) and half-life (t1/2) values were increased by 2°C and 7.8-folds, respectively. Molecular dynamics simulation showed reduced flexibility and additional chemical bonds in the region near the mutation site.

**Discussion:**

These results indicate that we obtained a Anman mutant that is more suitable for industrial application, and they also confirm that a combination of rational and semi-rational techniques is helpful for screening mutant sites.

## 1. Introduction

Plant cell walls consist of hemicellulose and cellulose, which are both abundant polysaccharides in nature (Katrolia et al., 2012). Mannans are the main components of hemicelluloses, are widely found in plant seeds and endosperm, and are formed by mannose linked *via* ß-1,4 glycosidic bonds (Singh et al., 2018; von Freiesleben et al., 2019). Mannans can be classified into pure mannans, galactomannans, glucomannans, and galactoglucomannans based on their composition (Wang et al., 2015). Locust bean gum (LBG) and guar gum are commonly mixed substrates for mannanase, and the ratio of pure mannose to galactose in LBG and guar gum is 4:1 and 2:1, respectively (Cao et al., 2018).

Endo-ß-mannanase (EC 3.2.1.78) is present in plants, animals, and microorganisms (Yamaura and Matsumoto, 1993; Zhou et al., 2014; Leerawatthanakun et al., 2022). It can be classified into glycoside hydrolase (GH) families 5, 26, and 113 based on its sequence (Lombard et al., 2014).^1^ But in recent years, it is also considered to be a part of the GH134 family (Li et al., 2020; Sun et al., 2021). Mannanase degrades mannan to mannooligosaccharide (MOS) by cleaving the mannan backbone, and the MOS promotes the growth of probiotic bacteria such as *Bifidobacterium adolescentis* in the mammalian gut (Lv et al., 2013; Kumar Suryawanshi and Kango, 2021). In addition, mannanase is also applied in the fields of dye decolorization, pulp bleaching, detergent enhancement, coffee viscosity reduction, and oil drilling (Chauhan et al., 2012; Li et al., 2014; Sakai et al., 2017; Wang et al., 2018; Guo et al., 2021). However, most of the above industrial applications are currently carried out in high-temperature environments, thus, heat-resistant mannanase is the preferred enzyme in the feed granulation process, saccharification, and fermentation processes (Turner et al., 2007; Bhalla et al., 2013). However, the optimum temperature for microbial-derived mannanase is generally 40–60r, thus, obtaining a strain of ß-mannanase with excellent thermostability can create significant benefits.

Directed evolution is a traditional technique for accelerating the evolution of enzyme molecules by mimicking natural environmental changes, but random mutations in genes tend to produce large libraries of mutants; therefore, obtaining an excellent mutation by directed evolution is time-consuming and costly (Qiu et al., 2021). Meanwhile, many rational methods for improving the thermostability of enzymes have been reported, such as the design of surface amino acid charges, N- or C-terminal enzyme modification, mutation-free energy calculation, and molecular dynamics simulation (Mahanta et al., 2015; Srivastava et al., 2016; Herrera-Zúñiga et al., 2019; Liu et al., 2021). Rigid flexibility sites (RFS) are one of the protein rational design strategies. They could efficiently screen for mutation sites and improve enzyme thermostability based on the theory that enzyme inactivation at high temperatures was caused by the unfolding of its flexible regions due to heat (Yu et al., 2017). Thus, the flexible regions must be identified and rigidified to maintain enzyme activity at high temperatures. The B-factor and Gibbs unfolding free energy change (ΔΔG) are commonly used screening parameters in the rational design. They reflect the mobility of an atom during nuclear magnetic resonance and the conformational stability of the mutant compared to its wild-type (WT) counterpart, respectively (Kellogg et al., 2011; Yuan et al., 2018). Molecular dynamics simulations, called “computational molecular microscopy,” are a well-established technique that can be applied to study the structural, energetic, and thermodynamic properties of various molecules at any temperature and pressure (Feng et al., 2019; Mazurek et al., 2021).

To the best of our knowledge, this is the first study to achieve heterologous expression of an acidic GH5 family ß-mannanase from *Aspergillus niger* (accession number: XP_001390707) in *Pichia pastoris*. Then, B-factor and ΔΔG were used to screen for five positive single-point mutants, all of which showed a slight improvement in thermostability. Furthermore, the obtained multipoint combinatorial mutant showed better thermostability. Finally, the mutational effects were studied using multiple sequence alignment and molecular dynamics simulation. As a result, the present study described a new acidic ß-mannanase with good heat resistance for industrial applications and showed a successful combination use of rational and semi-rational design in the screening of thermophilic enzymes.

## 2. Materials and methods

### 2.1. Enzymes, vectors, and materials

The ß-mannanase gene was synthesized by Shanghai Sangon after codon optimization. pPICZ vector (preserved in our laboratory) was used to form a pPICZ-Anman recombinant vector with Anman. *Escherichia coli* JM109 and *P. pastoris* X33 were kept in our laboratory for cloning and heterologous expression of mannanase, respectively. Restriction endonuclease and DNA polymerase were purchased from Takara (Dalian, China). PCR purification kit was purchased from GENEWIZ (Suzhou, China). LBG was purchased from Sigma (St. Louis, MO, USA) as substrate. The rest of the chemical reagents not specified were analytically pure.

### 2.2. Homologous modeling and calculation of ΔΔG

Because of the high protein sequence similarity between 3WH9 (PDB ID) and Anman (Huang et al., 2014), we selected 3WH9 as the template for Anman using the SWISS-MODEL server for homology modeling (Supplementary Figure 1). The ΔΔG of Anman calculated using the Discovery Studio 2019 software (Fu et al., 2017). Briefly, using the DeepView software to complement the missing side chains and residues, the Charmm36 force field was imparted to the protein, and ΔΔG was calculated under pH-dependent conditions. ΔΔG was calculated as shown in Equations (1) and (2):


(1) ΔΔG=foldingΔG-fldΔGunf

(2) ΔΔG=mutΔΔG-folding(mutant)ΔΔG(wildtype)folding

ΔΔG_folding_ indicates the Gibbs unfolding free energy change of WT or mutant in the folding and unfolding states, and the mutant is predicted to be stable if it possesses negative ΔΔG_mut_, and the opposite is predicted to be unstable.

### 2.3. Selection of mutation sites

The B-factor of wild-type mannanase Anman was calculated by ba2r,^2^ and we subsequently obtained the secondary structure information of Anman using the PDBsum database.^3^ To reduce the loss of enzyme activity caused by the mutation effect, the average distance between catalytic residues and ß-turns in loops was measured using Pymol. Subsequently, we further narrowed down the range of mutation sites according to the amino acid preference of ß-turns and then combined it with ΔΔG screening to obtain hotspots. Meanwhile, Anman was compared with ß-mannanase with 13 excellent thermostability by multiple sequence alignment to explore semi-rational design’s role in finding hotspots.

### 2.4. Enzyme expression and purification

The recombinant vectors of Anman and mutants were linearized by restriction endonuclease *Sal* al restriction endonuclease. The linear vector was transferred into *P. pastoris* X33 and incubated in yeast extract peptone dextrose (YPD) solid medium containing 100 mg/mL bleomycin (zeocin) at 30°C for 72 h. Colonies were picked and incubated in 30 Ml YPD liquid medium at 30°C and 220 rpm for 24 h, and cells were then collected by centrifugation at 8,000 rpm and transferred to 30 mL YP medium containing 0.5% methanol at 28°C and 220 rpm for 72 h. Methanol was added every 12 h to maintain its concentration at 0.5% for induction of enzyme production.

The induced fermentation broth was centrifuged at 4°C at 8,000 rpm, and the collected supernatant was dialyzed for 16 h in 0.1 M NaH_2_PO_4_-Na_2_HPO_4_ containing 20 mM NaCl (pH 5.5, buffer A) to remove salt and metal ions. Anion exchange chromatography was used for the purification of crude enzymes. Briefly, after pre-equilibrating the anion exchange column (Hi Trap™ 1 mL Q HP) with Buffer A, the dialyzed crude enzyme was loaded onto the column, followed by a linear gradient elution using Buffer A with Buffer B (Buffer A with 1 M NaCl), and the collection containing the enzyme activity was utilized for the subsequent study. The purification of the samples was analyzed by sodium dodecyl sulfate-polyacrylamide gel electrophoresis (SDS-PAGE) (Laemmli, 1970). The Bradford method was used to determine the protein concentration (Bradford et al., 1976).

### 2.5. Determination of mannanase activity

The determination of ß-mannanase activity was performed by the 3,5-dinitrosalicylic acid (DNS) method. Briefly, the purified enzyme solution and LBG were diluted and dissolved with 0.1 M acetic acid-sodium acetate buffer (pH 3.0, Buffer C), and the reaction system consisted of 80 μL of pure enzyme and 160 μL of LBG (0.3 mg/mL), incubated at 37°C for 30 min and then terminated by adding 200 μL of DNS. After boiling for 5 min, 560 μL of water was added to the reaction system, and the absorbance of the reaction solution at 540 nm was measured using a microplate reader. Under the same conditions, the absorbance of mannan oligosaccharides was used as the standard curve. One unit of ß-mannanase activity was defined as the amount of enzyme that releases 1 μmol of reducing sugar per minute under the above conditions. All experiments were performed in three parallel experiments.

### 2.6. Determination of biochemical properties of mannanase

The specific activity of mannanase at different temperatures (50–80°C) was determined using the method mentioned above, and the temperature with the highest specific activity was determined as its optimum temperature. Thermostability was determined by incubating mannanase at 70°C for different times (10–60 min) and then reacting with LBG at 37°C to determine the residual activity. Tris-HCl (pH 2.0) and acetic acid-sodium acetate (pH 3.0–7.0) were used to determine the optimum pH of mannanase, the specific activity of mannanase was measured at different pH conditions (2.0–7.0), and the pH with the highest specific activity was determined as the optimum pH.

To determine the *t*_1/2_ of mannanase, the enzyme was incubated at 70°C for different periods (5–90 min) and then reacted with LBG to determine its remaining specific activity. The kinetic parameters were determined using 1–10 mg/mL LBG as the substrate (concentration gradient of 1 mg/mL) with the pure enzyme at 37°C and pH 3.0 to determine specific activity. The *K*_*m*_ and *K*_cat_ parameters were obtained by non-linear fitting using the Origin 2017 software according to the specific activity and LBG concentration. All the above experiments were performed in three parallel experiments.

### 2.7. Molecular dynamics simulation

Molecular dynamics simulations of wild-type Anman and its mutants were performed using Gromacs 2018. Briefly, the missing atoms were added to the PDB file of Anman using the DeepView software before adding the Amberff14sb force field, followed by adding the TIP3P water model to the ortho-dodecagonal box and neutralizing the charge in the system with a sufficient amount of sodium ions. Next, the entire system was minimized using the fastest descent method (1,000 steps), followed by NVT and NPT simulations for 200 ns, respectively, using the LINCS algorithm to limit the heavy atoms and all bonds. The above processes were performed under periodic boundary conditions (PBC). Finally, the system was simulated at 370 K for 50 ns, with a trajectory saving interval of 2 fs. The parameters of root mean square deviation (RMSD), root mean square fluctuation (RMSF), and hydrogen bonds during the simulation were analyzed by the saved trajectory file, and VMD carried out the analysis of salt bridges.

### 2.8. Determination of melting temperature

The protein melting temperature (*T*_m_) represents the thermodynamic stability and reflects the conformational changes of the enzyme at high temperatures. In this study, *T*_m_ was determined using the Nano-Differential SCNAning Calorimeter (Nano-DSC, Waters, USA) instrument. Briefly, the pure enzyme was diluted to a concentration of 0.2 mg/mL with Buffer C and loaded into a capillary cell. The scanning temperature range was 20–100°C with a ramp rate of 1°C/min, and Buffer C was used as the control. The data were analyzed using the Nano Analyze software.

### 2.9. Constraint network analysis

Constraint network analysis (CNA) simulates the enzymes as body-and-bar networks, where the forces between atoms are replaced by bars. The number of bars represents the strength, and E_hb_ measures the magnitude of the energy contained in the force. CNA simulates a thermal unfolding process with increasing temperature, and the E_cut–hb_ decreases as the temperature increases. During the CNA simulation, there is a sudden decrease in the rigidity order parameter (*P*_∞_), which means that the network changes from rigid to flexible, just like the protein folding-unfolding transition, and the E_cut–hb_ is the phase transition point. The temperature of conformational transition (T) can be calculated by using Equation (3):


(3) T=-20K/(kcal⋅mol)-1⋅E+c⁢u⁢t-h⁢b300K

Constraint network analysis was accomplished on a web service.^4^ In this study, the range of E_hb_ was set from –0.1 to –0.6 kcal/mol, and the decreasing gradient of E_cut–hb_ was –0.1 kcal/mol.

## 3. Results and discussion

### 3.1. Determination of flexible regions

To identify the more flexible regions in Anman, the average B-factor of α-helices, β-sheets, and loops was calculated, which were 20.5, 17.7, and 21.4, respectively (Supplementary Figure 2). The regions with a higher B-factor level tended to contain more flexible residues; therefore, the present study focused on the mutation range in loop regions, and it has been also confirmed that the modification in loop regions could improve thermostability (Cheng et al., 2012). Due to the different flexibilities of the enzyme surface and internal loops, the B-factors of residues exposed on the surface vs. those buried in the interior were calculated as 24.1 and 18.2, respectively. Meanwhile, the calculated average B-factor of residues within 5 Å of the catalytic residue was only 14.8, suggesting that the protein interior was more stable than the surface. Thus, we narrowed the selection of mutation sites to loop regions located on the surface of Anman.

**FIGURE 1:**
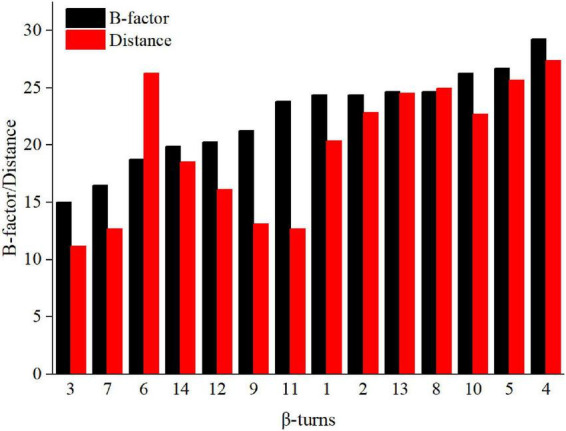
B-factor (black column) and average distance (red column) of loops in Anman.

There are many β-turns on loop regions, thus, we can design mutations based on the amino acid preferences on different residues (position i, i + 1, i + 2, i + 3) of β-turns (Supplementary Figure 3). In addition, proline has a higher frequency at position i + 1, it is the most rigid of all amino acids, and Xaa→Pro is beneficial for the improvement of thermostability (Wang et al., 2014; Land et al., 2019). Thus, the mutation range was further narrowed to β-turns of Anman (types as furth, and we prefer mutation to proline. The Pymol software was used to calculate the average distance of C_*a*_ for each β-turn from catalytic residues Glu168 and Glu276, and their correlation with the B-factor is shown in Figure 1. Except for β-turn 6, all other β-turns had a higher correlation in B-factor and distance from catalytic residues, and β-turns with a higher B-factor tended to be further away from catalytic residues. To obtain mutants that do not affect the catalytic efficiency, several regions (β-turn 3, 7, 9, 11) that were closer to the catalytic residues were excluded, and the remaining β-turns were used for the next round of screening.

### 3.2. Calculation of ΔΔG and mutation

**FIGURE 2:**
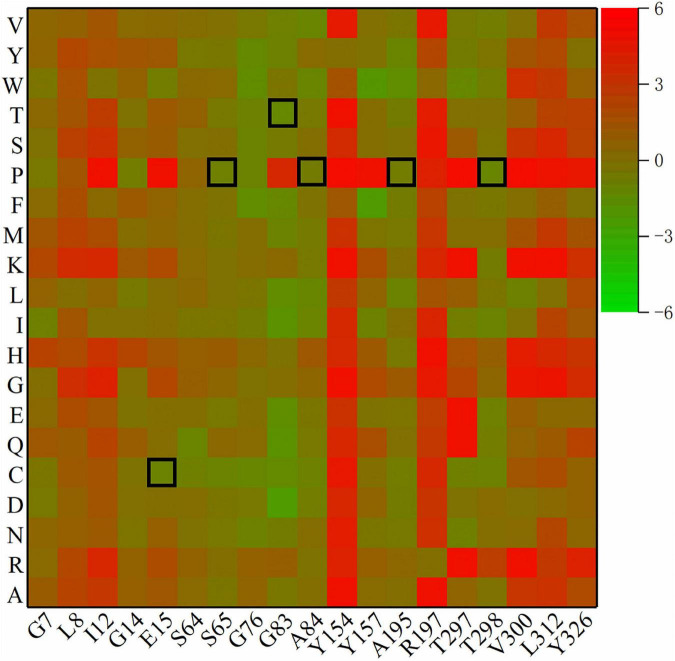
Heatmap of ΔΔG for mutants. Green means ΔΔG is negative and is predicted to be a stable mutation, while red means an unstable mutation, and the highlighted black squares indicate the mutation sites generated by this experiment.

The remaining β-turns were subjected to virtual saturation mutation using the Discovery Studio 2019 software (Fu et al., 2017), and their ΔΔG was calculated. The heatmap in Figure 2 shows the ΔΔG for 380 mutants, and negative values represent mutations that are predicted to be stable. Since the calculation error for ΔΔG was about 0.5 kJ/mol, a total of 315 mutants were predicted to be neutral or deleterious (their ΔΔG > –0.5 kJ/mol). Some residues with high ΔΔG values (including I12, Y154, R197, V300, and L312) were excluded. Finally, six single-point mutations (E15C, S65P, G83T, A84P, A195P, and T298P) were selected using a β-turn preference for flexible modifications. D114A and E222T (predicted to be negative mutations) were selected for use as the controls due to their higher ΔΔG values (0.52 and 2.22 kJ/mol, respectively) and closer distances from catalytic residues (11.9 and 10.5 Å, respectively).

### 3.3. Construction of mutants and heterologous expression

Using the wild-type Anman gene as the template, all mutants were generated using polymerase chain reaction point mutation and verified *via* DNA sequencing. The primers used for all mutants are shown in Supplementary Table 1. Anman and positive mutants were overexpressed using *P. pastoris* X33 and then purified and verified by SDS-PAGE. The crude enzyme in lane 2 had an inconspicuous protein band at 80 kDa, while all of the remaining lanes showed a distinct single band at 47 kDa, which was inconsistent with the calculated molecular weights (37.1 kDa) due to the processing of glycosylation modification of Anman before its extracellular secretion by yeast cells (Supplementary Figure 4).

### 3.4. Biochemical properties of Anman and mutants

**FIGURE 3:**
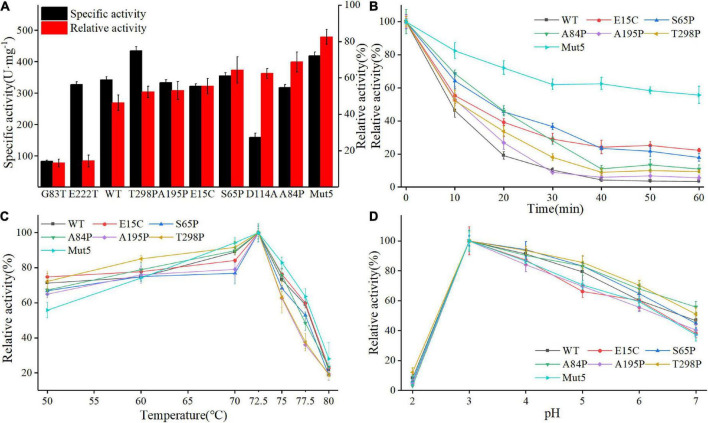
Biochemical properties of Anman and Mut5. **(A)** Specific activity (black column) and relative activity (red column) after 10 min incubation at 70°C of Anman and mutants, and relative activity was obtained by the ratio of residual activity after 10 min of incubation at 70°C to the initial activity without incubation. **(B)** Relative activity of Anman and mutants after incubation at 70°C for 0–60 min. **(C)** The optimal temperature of Anman and mutants. **(D)** Optimal pH for Anman and mutants.

Figure 3A shows the specific activity (black bars) and thermostability (red bars) of Anman and all mutants. Most of the mutants (excluding G83T and D114A) showed no significant decrease in a specific activity, which was due to the long distance between the mutation site and catalytic residue. As expected, the thermostability of mostly single-point mutants was improved, and A84P is the best-performing single-point mutant (from 46.2 to 68.7%). However, the relative activity of G83T was only 0.28-fold the level of Anman, which was an unexpected result. Finally, five single-site mutants with improved thermostability and no significant reduction in specific activity were combined to obtain the combinatorial mutant Mut5 (E15C/S65P/A84P/A195P/T298P). Mut5 had a 0.2-fold higher specific activity and a 0.7-fold higher thermostability than Anman (Table 1). It retained 82.4% of its activity after 10 min of incubation at 70°C, which meant that the obtained thermophilic β-mannanase mutant had a significantly improved thermostability without the loss of enzyme activity.

**TABLE 1:** Kinetic and thermodynamic parameters of Anman and mutants.

	Specific activity (U⋅mg^–1^)	Relative activity^†^ (%)	*K*_m_ (mg⋅mL^–1^)	*K*_cat_ (s^–1^)	*K*_cat_/*K*_m_ (mL⋅mg^–1^s^–1^)	*t*_1/2_ (min)	*T*_m_ (°C)
Anman	341.5	46.2	5.6	1075.7	191.0	8.3	75.7
E15C	321.5	55.43	6.7	1114.4	164.6	13.2	76.1
S65P	355.1	64.3	6.0	1039.1	171.1	18.7	76.4
A84P	316.6	68.7	6.4	1044.1	161.6	18.9	76.4
A195P	333.6	53.0	5.2	1155.2	221.3	11.3	74.7
T298P	434.0	52.2	5.9	1095.0	184.6	11.6	76.5
Mut5	418.3	82.4	5.2	1252.7	239.0	73.4	77.7

^†^Enzyme relative activity of wild-type and mutants after incubation at 70°C for 10 min.

To further investigate the performance of the mutant and Anman at high temperatures, their residual activity was measured after incubation at 70°C for 0–60 min (Figure 3B). The single-point mutant showed a slight increase over Anman, and E15C became the single-point mutant with the highest residual activity after 60 min of incubation, still retaining 22.3% of its specific activity, while Anman was almost completely inactivated. The combined mutant Mut5 had a remaining activity of 55.73%, which was significantly higher than that of Anman throughout the incubation process, indicating that Mut5 was more suitable for long-term function and industrial production at high temperatures than Anman.

Anman and all mutants showed the highest activity at 72.5°C, with no significant change in the specific activity below 70°C. But, the activity of Anman and mutants decreased dramatically when the temperature was increased to 75°C (Figure 3C). The mutation effect did not increase the optimal temperature of β-mannanase, which implies that it is more challenging to engineer thermophilic enzymes than mesophilic enzymes. The optimum pH of both Anman and the mutant was 3.0, which is close to the acidic environment of the animal stomach, suggesting that Anman and Mut5 can be used as feed additives in the breeding industry (Figure 3D).

### 3.5. Multiple sequence alignment

To further explore the reasons for the decrease in enzyme activity or thermostability of G83T, D114A, and E222T, the protein sequence of Anman was aligned with 13 GH5 family thermostable β-mannanases, and their derived strains are shown in Supplementary Table 2. The sequence conservation of E15, S65, A195, and T298 was less than 50%, G83 and E222 reached 92.3%, while D114 was up to 100% (Supplementary Figure 5). The comparison results indicated that G83, D114, and E222 were highly conserved residues, and their mutations occurred against the natural evolution, which was why they had negative mutation effects. The above results also demonstrated that relying on rational design alone could not completely exclude negative mutants, and introducing other methods in the screening process will be more beneficial in finding mutation sites. For example, Liu generated 44 mutants by modifying the surface-charged amino acids of ManAK, but only three mutants performed well (Liu et al., 2021).

### 3.6. Kinetic parameters and thermodynamic stability

To investigate the effect of mutation on the kinetics of Anman, the kinetic parameters of Anman and mutants were measured using 1–10 mg/ml LBG as the substrate (Table 1). Mostly, mutants had a similar *K*_m_ as Anman, but E15C reached 6.7 mg/mL. Mut5 showed a slight increase in *K*_cat_, indicating that it had a higher affinity for LBG than Anman. There was no significant difference in catalytic efficiency *K*_cat_/*K*_m_ for single-point mutants, but Mut5 was 0.25-fold higher than Anman, reaching 239 mL/mg s (Supplementary Figure 6). The above results revealed that the mutation did not impair the catalytic ability of Anman for LBG, which further verified that mutations farther away from the catalytic residues had a lower effect on enzyme activity and that it could be one of the conditions used for screening thermostable mutants.

The *T*_m_ for Anman was 75.7°C, which was close to its optimal temperature, and this was likely due to the protective effect of LBG on the catalytic pocket of β-mannanase. Except for A195P, the *T*_m_ for other mutants was higher than that for Anman, but the Δ*T*_m_ for all single-point mutants was less than 1°C, and the *T*_m_ value for Mut5 was only improved by 2°C compared to that for Anman (Supplementary Figure 7). Anman only had a 1.2% residual activity at 75°C, while Mut5 retained 36.0% activity (Supplementary Figure 8), and both Anman and Mut5 were completely inactivated when the temperature reached 80°C. This property made Mut5 more suitable for high-temperature conditions in industrial applications.

The *t*_1/2_ was obtained by incubating at 70°C for different amounts of time. The *t*_1/2_ for A84P was the highest of all single-point mutants and 1.2-fold higher than that for Anman. Although the *t*_1/2_ for S65P and A84P was significantly higher compared to that for Anman, Mut5 was 7.8-fold higher than that for Anman, reaching 73.4 min, and this phenomenon was caused not only by epistatic effects but also by the modification of most of the flexible sites of Mut5. When there are multiple flexible sites on the surface, a single-point mutation often cannot prevent the process of thermal inactivation; therefore, if multiple or all flexible sites on the surface can be rigidified, enzyme thermostability will be dramatically enhanced, which is why Mut5 performed much better than the single-point mutants.

### 3.7. CNA and MD simulation

**FIGURE 4:**
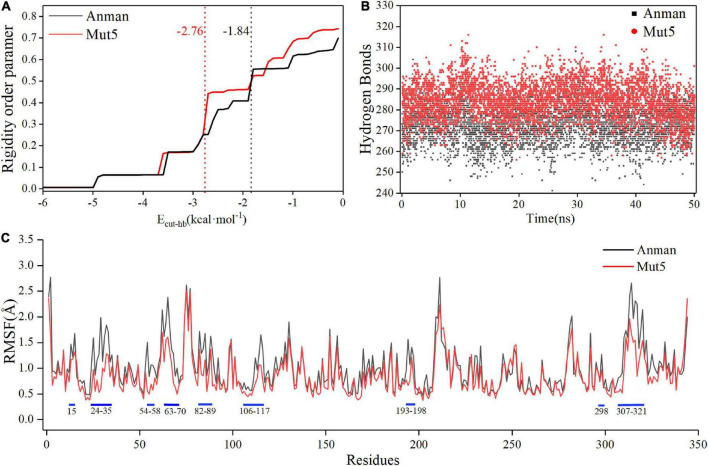
The constraint network analysis and molecular dynamics simulation of Anman (black) and Mut5 (red). **(A)** *P*_∞_ changes during thermal simulation, and Anman and Mut5 change from rigid to flexible at E_cut–hb_ of –1.84 and –2.76 kcal⋅mol^– 1^, respectively. **(B)** The number of hydrogen bonds of Anman and Mut5 during MD simulation. **(C)** RMSF of Anman and Mut5 at 370 K. Higher RMSF indicates more unstable residues, and the blue sticks represent the corresponding regions.

**FIGURE 5:**
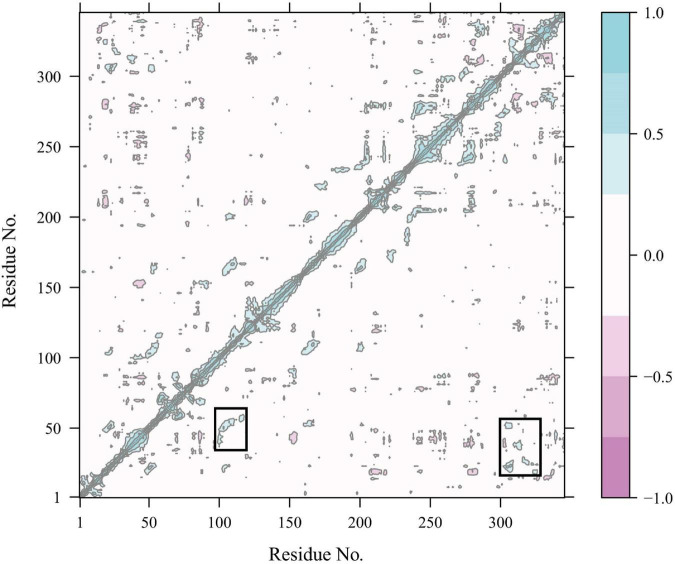
Dynamics cross-correlation map of C_α_ atoms in Anman. The white region represents residue motion without correlation; the purple region (cyan region) represents residue motion with correlation and in the same direction (opposite direction), and the darker color represents a stronger correlation.

Constraint network analysis can be used to visually compare the overall rigidity of Anman and Mut5. The *P*_∞_ of Anman and Mut5 decreased sharply when E_cut–hb_ was –1.84 and –2.76 kcal/mol, respectively (Figure 4A). The simulated temperatures were 336.8 and 355.2 K, according to Equation (3), which were consistent with the previous experimental results for *T*_m_ values. This also meant that Anman was inactivated earlier than Mut5. To further investigate the local and global stability of mannanase, a 50-ns kinetic simulation was carried out. It showed that the RMSD for Anman was usually higher than that for Mut5 (Supplementary Figure 9), and Mut5 had more hydrogen bonds for the first 45 ns (Figure 4B). These results indicated that the flexible engineered Mut5 was more stable than Anman in global conformation and more resistant to unfolding at high temperatures. MD simulation was also used to compare the local conformational stability of Anman and Mut5. The RMSF for all five mutation sites was reduced to various degrees, indicating that the mutation effect increased the rigidity of these sites (Figure 4C). The RMSF values in the region near the P65, P84, and P195 mutation sites were substantially reduced because the pyrrolidine proline ring did not only make the C_*a*_ and N atoms of the backbone more rigid in order to not rotate easily but also reduce the conformational flexibility of the nearby residues (Zhu et al., 2021). In addition, regions 24–35, 54–58, 106–117, and 307–321 also significantly reduced RMSF in the absence of mutations, probably because the mutation effect caused rearrangement of the internal force network through long-distance perturbation pathways (El Harrar et al., 2021). To investigate this phenomenon, the dynamic cross-correlation matrices (DCCMs) were calculated for Anman (Figure 5). There was a prominent cyan spot in the regions 54–58 and 106–117, which means that the atomic motions in these two regions were correlated and moved in the same direction, so their RMSF rise and fall trends should also be the same. In addition, region 54–58 was also spatially distributed adjacent to region 106–117, and there are many hydrogen bonds between the two regions; therefore, it is reasonable that the mutation causes a decrease in flexibility in both regions. The same process occurs in regions 24–35 and 307–321. The motion correlation of residues can be further used for the trade-off between thermostability and activity (Yu and Dalby, 2018).

### 3.8. Molecular force network analysis

**FIGURE 6:**
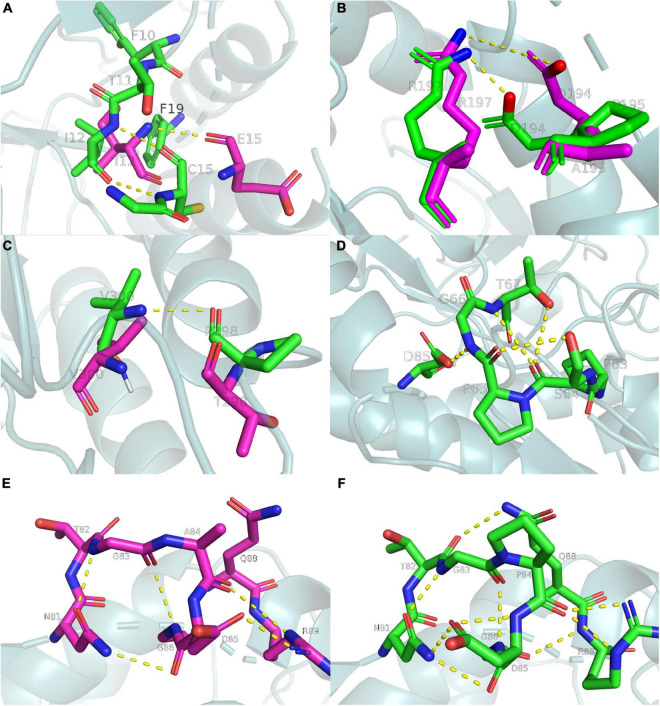
Ribbon diagrams showing the chemical bonds of Anman and Mut5, yellow dashed lines represent chemical bonds, purple and green main chains represent Anman and Mut5, respectively. **(A)** The E15C mutant forms additional hydrogen bonds and enhances hydrophobic interactions. **(B)** The A195P mutant increases the probability of salt bridge formation. **(C)** The T298P mutant forms an additional hydrogen bond. **(D)** The S65P mutant forms additional hydrogen bonds. **(E,F)** The A84P mutant forms additional hydrogen bonds. Structure visualization was performed using the pymol software.

The present study analyzed the chemical bonds within 5 Å of each mutation site (Figure 6). Several hydrophobic residues were located around E15, and the hydrophobicity of cysteine was much higher than that of glutamate. Thus, the E15C mutant site formed hydrophobic interactions with the surrounding residues to stabilize the region. A similar phenomenon was observed in lipolytic enzymes (Gallardo et al., 2010). The N atom of C15 also formed an additional hydrogen bond with the O atom of I12 (Figure 6A), which increased the thermostability of the E15C mutant. Even though A195P did not form more hydrogen bonds than Anman, D194 formed a salt bridge with R197 in A195P due to the structural perturbation caused by the mutation. To verify this phenomenon, the number of frames forming a salt bridge was divided by the total number of frames in the MD simulation. As a result, the probability of forming a D194-R197 salt bridge increased from 51.1 to 91.1%, and the distance between the OD2 atom of D194 and the NH2 atom of R197 decreased from 6.3 to 3.5 Å. Therefore, the improved thermostability of A195P was due to the formation of a salt bridge (Figure 6B). For the T298P mutant, no hydrogen bonds formed since the angle between the O atom of T298 and the N and H atoms of V300 was lower than 120°. In comparison, the mutated proline changed the local conformation and formed hydrogen bonds between P298 and V300, thereby reducing the flexibility of the local region (Figure 6C).

There were no hydrogen bonds near the S65 region of Anman, and there were four hydrogen bonds in S65P. The O atom of S64 formed two hydrogen bonds with the N and OG1 atoms of T67 (Figure 6D), which made the unfolding of the S65P region require more heat. Therefore, S65P did not easily deactivate at high temperatures. The same happens in the A84P region (Figures 6E, F), where the NE2 atom of Q88 approached the T82 residue to form an additional hydrogen bond, and the OD1 atom of D85 and the O atom of P84 formed two hydrogen bonds. A situation where one atom becomes an acceptor for two hydrogen bonds can strengthen the binding between residues. In addition, the S65P and A84P mutation sites were relatively close to each other in space, and the mutation effects could be transmitted between them (Dinmukhamed et al., 2020). Therefore, the force network between the S65P and A84P regions could be significantly changed or even redistributed, and the mutational effect resulting from structural rearrangement may also have a positive epistatic effect, which may account for the significantly reduced RMSF of residues near the two regions.

## 4. Conclusion

In conclusion, β-mannanase derived from *A. niger* CBS513.88 was characterized and its biochemical properties were measured. The combined mutant Mut5 had an apparent increase in *t*_1/2_, which was 7.8-fold higher than that of Anman. Meanwhile, the *T*_m_ of Mut5 was also increased by 2while, the ed. The combined mutant Mut5 had to determine that Mut5 had more global and local stability. Additional chemical bonds were generated near the corresponding mutation sites, which is also the molecular mechanism behind the better Mut5 thermostability compared to that of Anman. The present study yielded a β-mannanase mutant that was more suitable for industrial applications. The RFS strategy also provided an effective way for screening enzyme hotspots.

## Data availability statement

The original contributions presented in this study are included in the article/Supplementary material, further inquiries can be directed to the corresponding authors.

## Author contributions

DW was in charge of the total design of this study and participated the statistical analysis. ST carried out the study and drafted the manuscript. NL and TG contributed to the revision of the overall idea of the manuscript and the addition of the experiments. XT, PZ, PC, and XY collected the important background information. All authors read the final manuscript and approved the submission.

## Footnotes

1
http://www.cazy.org/

2
http://sobereva.com/32

3
http://www.ebi.ac.uk/pdbsum/

4
https://cpclab.uni-duesseldorf.de/cna/

## References

[B1] Bhalla A. Bansal N. Kumar S. Bischoff K. M. Sani R. K. (2013). Improved lignocellulose conversion to biofuels with thermophilic bacteria and thermostable enzymes. *Bioresour. Technol.* 128 751–759. 10.1016/j.biortech.2012.10.145 23246299

[B2] Bradford M. Bradford M. Bradford M. M. Bradford M. (1976). A rapid and sensitive method for the quantification of microgram quantities of protein using the principle of protein-dye binding. *Anal. Biochem.* 72 248–254. 10.1006/abio.1976.9999 942051

[B3] Cao L. Liu W. Wang L. (2018). Developing a green and edible film from *Cassia gum*: The effects of glycerol and sorbitol. *J. Clean. Prod.* 175 276–282. 10.1016/j.jclepro.2017.12.064

[B4] Chauhan P. S. Puri N. Sharma P. Gupta N. (2012). Mannanases: Microbial sources, production, properties and potential biotechnological applications. *Appl. Microbiol. Biotechnol.* 93 1817–1830. 10.1007/s00253-012-3887-5 22314515

[B5] Cheng Y. S. Ko T. P. Huang J. W. Wu T. H. Lin C. Y. Luo W. (2012). Enhanced activity of *Thermotoga maritima* cellulase 12A by mutating a unique surface loop. *Appl. Microbiol. Biotechnol.* 95 661–669. 10.1007/s00253-011-3791-4 22170108

[B6] Dinmukhamed T. Huang Z. Liu Y. Lv X. Li J. Du G. (2020). Current advances in design and engineering strategies of industrial enzymes. *Syst. Microbiol. Biomanuf.* 1 15–23. 10.1007/s43393-020-00005-9

[B7] El Harrar T. Frieg B. Davari M. D. Jaeger K. E. Schwaneberg U. Gohlke H. (2021). Aqueous ionic liquids redistribute local enzyme stability via long-range perturbation pathways. *Comput. Struct. Biotechnol. J.* 19 4248–4264. 10.1016/j.csbj.2021.07.001 34429845 PMC8355836

[B8] Feng J. Chen J. Selvam B. Shukla D. (2019). Computational microscopy: Revealing molecular mechanisms in plants using molecular dynamics simulations. *Plant Cell* 31:1319. 10.1105/tpc.119.tt1219 31822566 PMC6925007

[B9] Fu Y. Alashi A. M. Young J. F. Therkildsen M. Aluko R. E. (2017). Enzyme inhibition kinetics and molecular interactions of patatin peptides with angiotensin I-converting enzyme and renin. *Int. J. Biol. Macromol.* 101, 207–213. 10.1016/j.ijbiomac.2017.03.054 28300587

[B10] Gallardo O. Pastor F. I. Polaina J. Diaz P. Lysek R. Vogel P. (2010). Structural insights into the specificity of Xyn10B from *Paenibacillus barcinonensis* and its improved stability by forced protein evolution. *J. Biol. Chem.* 285 2721–2733. 10.1074/jbc.M109.064394 19940147 PMC2807328

[B11] Guo Z. Yi D. Hu B. Shi Y. Xin Y. Gu Z. (2021). The alteration of gut microbiota by bioactive peptides: A review. *Syst. Microbiol. Biomanuf.* 1 363–377. 10.1007/s43393-021-00035-x

[B12] Herrera-Zúñiga L. D. Millán-Pacheco C. Viniegra-González G. Villegas E. Arregui L. Rojo-Domínguez A. (2019). Molecular dynamics on laccase from *Trametes versicolor* to examine thermal stability induced by salt bridges. *Chem. Phys.* 517 253–264. 10.1016/j.chemphys.2018.10.019

[B13] Huang J. W. Chen C. C. Huang C. H. Huang T. Y. Wu T. H. Cheng Y. S. (2014). Improving the specific activity of beta-mannanase from *Aspergillus niger* BK01 by structure-based rational design. *Biochim. Biophys. Acta* 1844 663–669. 10.1016/j.bbapap.2014.01.011 24480109

[B14] Katrolia P. Zhou P. Zhang P. Yan Q. Li Y. Jiang Z. (2012). High level expression of a novel β-mannanase from *Chaetomium* sp. Exhibiting efficient mannan hydrolysis. *Carbohydr. Polym.* 87 480–490. 10.1016/j.carbpol.2011.08.008 34662993

[B15] Kellogg E. H. Leaver-Fay A. Baker D. (2011). Role of conformational sampling in computing mutation-induced changes in protein structure and stability. *Proteins* 79 830–838. 10.1002/prot.22921 21287615 PMC3760476

[B16] Kumar Suryawanshi R. Kango N. (2021). Production of mannooligosaccharides from various mannans and evaluation of their prebiotic potential. *Food Chem.* 334:127428. 10.1016/j.foodchem.2020.127428 32688173

[B17] Laemmli U. K. (1970). Cleavage of structural proteins during the assembly of the head of bacteriophage T4. *Nature* 227 680–685. 5432063 10.1038/227680a0

[B18] Land H. Campillo-Brocal J. C. Svedendahl Humble M. Berglund P. (2019). B-factor guided proline substitutions in chromobacterium violaceum amine transaminase: Evaluation of the proline rule as a method for enzyme stabilization. *Chembiochem* 20 1297–1304. 10.1002/cbic.201800749 30637901 PMC6593452

[B19] Leerawatthanakun S. Charoenwongpaiboon T. Klaewkla M. Chunsrivirot S. Sirirak J. Sriwitool T. E. (2022). High surfactant-tolerant beta-mannanase isolated from *Dynastes hercules* larvae excrement, and identification of its hotspot using site-directed mutagenesis and molecular dynamics simulations. *Enzyme Microb. Technol.* 154:109956. 10.1016/j.enzmictec.2021.109956 34871822

[B20] Li J. Wei X. Tang C. Wang J. Zhao M. Pang Q. (2014). Directed modification of the *Aspergillus usamii* β-mannanase to improve its substrate affinity by in silico design and site-directed mutagenesis. *J. Ind. Microbiol. Biotechnol.* 41 693–700. 10.1007/s10295-014-1406-7 24493565

[B21] Li Y. X. Liu H. J. Shi Y. Q. Yan Q. J. You X. Jiang Z. Q. (2020). Preparation, characterization, and prebiotic activity of manno-oligosaccharides produced from cassia gum by a glycoside hydrolase family 134 β-mannanase. *Food Chem.* 309:125709. 10.1016/j.foodchem.2019.125709 31708343

[B22] Liu Z. Fu X. Yuan M. Liang Q. Zhu C. Mou H. (2021). Surface charged amino acid-based strategy for rational engineering of kinetic stability and specific activity of enzymes: Linking experiments with computational modeling. *Int. J. Biol. Macromol.* 182 228–236. 10.1016/j.ijbiomac.2021.03.198 33831449

[B23] Lombard V. Golaconda Ramulu H. Drula E. Coutinho P. M. Henrissat B. (2014). The carbohydrate-active enzymes database (CAZy) in 2013. *Nucleic Acids Res.* 42 D490–D495. 10.1093/nar/gkt1178 24270786 PMC3965031

[B24] Lv J. Chen Y. Pei H. Yang W. Li Z. Dong B. (2013). Cloning, expression, and characterization of β-mannanase from *Bacillus subtilis* MAFIC-S11 in *Pichia pastoris*. *Appl. Biochem. Biotechnol.* 169 2326–2340. 10.1007/s12010-013-0156-8 23446982

[B25] Mahanta P. Bhardwaj A. Kumar K. Reddy V. S. Ramakumar S. (2015). Structural insights into N-terminal to C-terminal interactions and implications for thermostability of a (β/α)8-triosephosphate isomerase barrel enzyme. *FEBS J.* 282 3543–3555. 10.1111/febs.13355 26102498

[B26] Mazurek A. H. Szeleszczuk L. Gubica T. (2021). Application of molecular dynamics simulations in the analysis of cyclodextrin complexes. *Int. J. Mol. Sci.* 22:9422. 10.3390/ijms22179422 34502331 PMC8431145

[B27] Qiu Y. Hu J. Wei G. W. (2021). Cluster learning-assisted directed evolution. *Nat. Comput. Sci.* 1 809–818. 10.1038/s43588-021-00168-y 35811998 PMC9267417

[B28] Sakai K. Mochizuki M. Yamada M. Shinzawa Y. Minezawa M. Kimoto S. (2017). Biochemical characterization of thermostable β-1,4-mannanase belonging to the glycoside hydrolase family 134 from *Aspergillus oryzae*. *Appl. Microbiol. Biotechnol.* 101 3237–3245. 10.1007/s00253-017-8107-x 28105485

[B29] Singh S. Singh G. Arya S. K. (2018). Mannans: An overview of properties and application in food products. *Int. J. Biol. Macromol.* 119 79–95. 10.1016/j.ijbiomac.2018.07.130 30048723

[B30] Srivastava P. K. Appu Rao G. A. Kapoor M. (2016). Metal-dependent thermal stability of recombinant endo-mannanase (ManB-1601) belonging to family GH 26 from *Bacillus* sp. CFR1601. *Enzyme Microb. Technol.* 84 41–49. 10.1016/j.enzmictec.2015.12.010 26827773

[B31] Sun D. Zhang J. Li C. Wang T. F. Qin H. M. (2021). Biochemical and structural characterization of a novel thermophilic and acidophilic β-mannanase from *Aspergillus calidoustus*. *Enzyme Microb. Technol.* 150:109891. 10.1016/j.enzmictec.2021.109891 34489044

[B32] Turner P. Mamo G. Karlsson E. N. (2007). Potential and utilization of thermophiles and thermostable enzymes in biorefining. *Microb. Cell Fact.* 6:9. 10.1186/1475-2859-6-9 17359551 PMC1851020

[B33] von Freiesleben P. Moroz O. V. Blagova E. Wiemann M. Spodsberg N. Agger J. W. (2019). Crystal structure and substrate interactions of an unusual fungal non-CBM carrying GH26 endo-β-mannanase from *Yunnania penicillata*. *Sci. Rep.* 9:2266. 10.1038/s41598-019-38602-x 30783168 PMC6381184

[B34] Wang C. Luo H. Niu C. Shi P. Huang H. Meng K. (2015). Biochemical characterization of a thermophilic β-mannanase from *Talaromyces leycettanus* JCM12802 with high specific activity. *Appl. Microbiol. Biotechnol.* 99 1217–1228. 10.1007/s00253-014-5979-x 25104029

[B35] Wang K. Luo H. Tian J. Turunen O. Huang H. Shi P. (2014). Thermostability improvement of a *Streptomyces xylanase* by introducing proline and glutamic acid residues. *Appl. Environ. Microbiol.* 80 2158–2165. 10.1128/AEM.03458-13 24463976 PMC3993148

[B36] Wang X. C. You S. P. Zhang J. X. Dai Y. M. Zhang C. Y. Qi W. (2018). Rational design of a thermophilic β-mannanase from *Bacillus subtilis* TJ-102 to improve its thermostability. *Enzyme Microb. Technol.* 118 50–56. 10.1016/j.enzmictec.2018.07.005 30143199

[B37] Yamaura I. Matsumoto T. (1993). Purification and some properties of endo-β-1,4 mannanase from a *Mud snail* Pomacea insulars. *Biosci. Biotech. Biochem.* 57 1316–1319. 10.1271/bbb.57.1316 7764016

[B38] Yu H. Dalby P. A. (2018). Exploiting correlated molecular-dynamics networks to counteract enzyme activity–stability trade-off. *Proc. Natl. Acad. Sci. U.S.A.* 115 12192–12200. 10.1073/pnas.1812204115 30530661 PMC6310800

[B39] Yu H. Yan Y. Zhang C. Dalby P. A. (2017). Two strategies to engineer flexible loops for improved enzyme thermostability. *Sci. Rep.* 7:41212. 10.1038/srep41212 28145457 PMC5286519

[B40] Yuan Z. Bailey T. L. Teasdale R. D. (2018). Prediction of protein flexibility profile. *Funct. Bioinform.* 58 905–912. 10.1002/prot.20375 15645415

[B41] Zhou P. Liu Y. Yan Q. Chen Z. Qin Z. Jiang Z. (2014). Structural insights into the substrate specificity and transglycosylation activity of a fungal glycoside hydrolase family 5 β-mannosidase. *Acta Crystallogr. D Biol. Crystallogr.* 70 2970–2982. 10.1107/S1399004714019762 25372687

[B42] Zhu C. Chen Y. Isupov M. N. Littlechild J. A. Sun L. Liu X. (2021). Structural insights into a novel esterase from the east pacific rise and its improved thermostability by a semirational design. *J. Agric. Food Chem.* 69 1079–1090. 10.1021/acs.jafc.0c06338 33445864

