# Supplementary material for: Thermostability modification of β-mannanase from Aspergillus niger via flexibility modification engineering
Source: Front Microbiol. 2023 Feb 20;14:1119232. doi: 10.3389/fmicb.2023.1119232 (PMC9986629; doi:10.3389/fmicb.2023.1119232)

Supplemental Information

Thermostability Modification of β-mannanase from *Aspergillus niger* via flexibility modification engineering

Shundong Tan, Xiumei Tao, Pu Zheng, Pengcheng Chen, Dan Wu*, Tiecheng Gao*, Ning Li

The Key Laboratory of Industrial Biotechnology, Ministry of Education, School of Biotechnology, Jiangnan University, Wuxi 214122, China

*Corresponding author.

Dan Wu:

Address: School of Biotechnology, Jiangnan University, 1800 Lihu Road, Wuxi 214122, China

Tel/fax: +86 510 8591 8156

e-mail: wudan@jiangnan.edu.cn

Tiecheng Gao:

Address: Guangzhou Puratos Food Co. Ltd., Guangzhou 511400, China

e-mail: rgao@puratos.com

Table S1, primers used for gene cloning and site-directed mutagenesis, underlines represent mutation sites.

	Primer
E15C	F1: TGTACTGGTTACTTCGCTGGTAC
	R1: GCGAAGTAACCAGTACAACCGTCAATGGTGAATTGC
S65P	F1: CCAGGTACTGTTTGGTATCAATTGCAC
	R1: TACCAAACAGTACCTGGAGATGGCTGAGAAGTAAC
G83T	F1: CTACTATTAACACCACTGCTGATGGATTGCAAAGATT
	R1: GTGGTGTTAATAGTAGATTTACCATCTTGG
A84P	F1: CTATTAACACCGGTCCAGATGGATTGCAAAGATTGGATTAC
	R1: TGGACCGGTGTTAATAGTAGATTTACCAT
D114A	F1: CTTATGGTGGTATGTCTGCTTATG
	R1: ACATACCACCATAAGCAGTCCAGTAGTTAACAAAGTTGAT
A195P	F1: TTAAGGGTTTGGATCCAGATCGTATGGTTTGTATTGGTGAC
	R1: TGGATCCAAACCCTTAATAAACTTAGAGG
E222T	F1: ACTGGATTGAACTTCACTATGAACTTG
	R1: GTGAAGTTCAATCCAGTGGAGAACTGGTATGGGTAAGAAC
T298P	F1: CTGCTTTGTCCACTCCAGGTGTTGGTGCTGATTTGT
	R1: TGGAGTGGACAAAGCAGTCTTTT

Table S2, Mannanase for sequence alignment.

mannanase	T_{opt}	pH_{opt}	Host strains	Accession number
man5XZ3	80	5.0	*Aspergillus nidulans* XZ3	AGG69666
ManBK	80	4.5	*Aspergillus niger* BK01	ACJ06979
Anman5A	70	3.5	*Aspergillus niger* LW-1	AEY76082
Asman	70-75	3.5-4.5	*Aspergillus sp.* T16	AHA38074
AuMan5A	70	3.5	*Aspergillus usamii* YL-01-78	ADZ99027
MAN5	70	6.0	*Bispora antennata* CBS 126.38	AFJ68087
Man5A	85–90	4.0	*Talaromyces leycettanus* JCM12802	AJF11663
rPoMan5A	80	4.0	*Penicillium oxalicum* GZ-2	AGW24296
man5C6	70	4.5	*Penicillium sp.* C6	AEV41143
Man5C1	70	4.0	*Penicillium pinophilum* C1	AEV40667
bMan2	69	5.2	*Myceliophthora thermophila* C1	AFJ59924
man5XZ7	75	5.0	*Thielavia arenaria* XZ7	AGG69667
ThMan5A	70	6.0	*Trichoderma harzianum* MGQ2	AGH62580

Figure S1, The three-dimensional structure of Anman. Red represents the catalytic residues, purple represents the mutation sites, green represents the amino acids within 5 Å of each mutation site, and the yellow dashed line represents the distance between the mutation site and the catalytic residues.

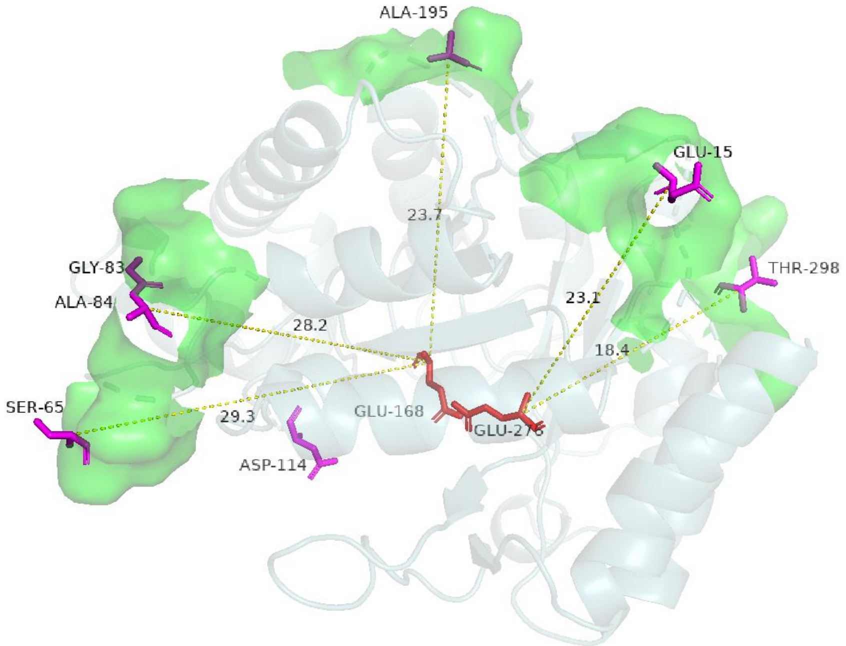

Figure S2, B-factor in different regions of Anman. "Exposed" represents residues that are on the surface of the protein and accessible to the solvent, otherwise "Buried", and "E168/E276" represent residues within 5 Å of the catalytic residue.

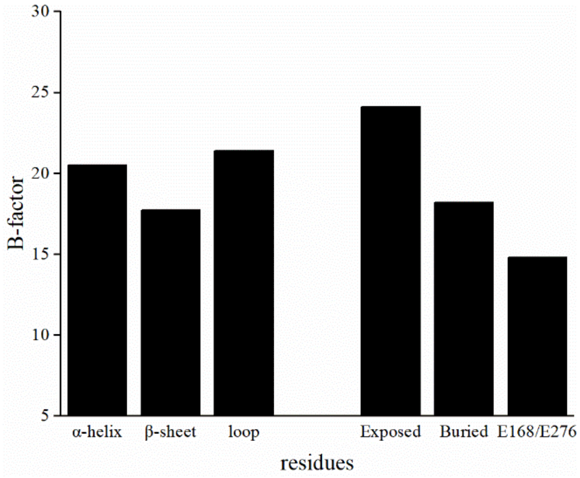

Figure S3, Amino acid preference of the β-turns (type I and II).

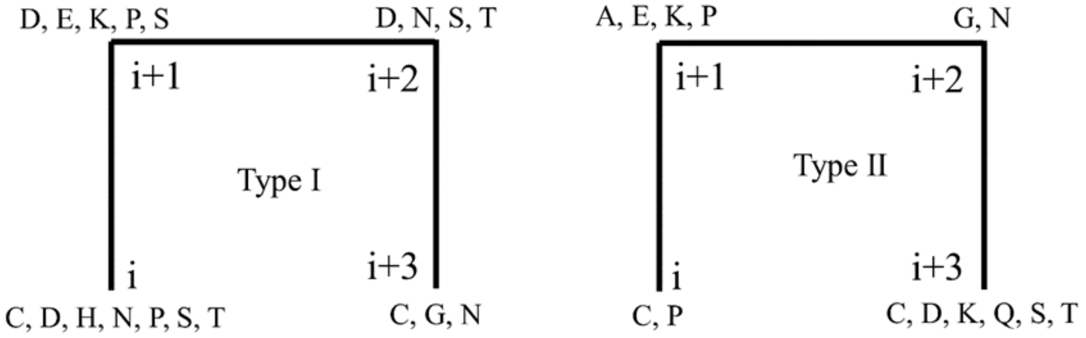

Figure S4, SDS-PAGE analysis of purified wild-type Anman and its mutants. Lane M represents marker. Lane 1 represents the unpurified supernatant. Lanes 2-8 represent purified Anman, E15C, S65P, A84P, A195P, T298P and Mut5, respectively.

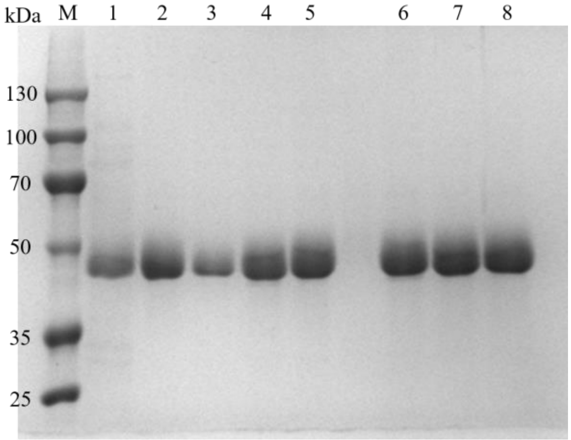

Figure S5, Amino acid sequence alignment of Anman and β-mannanase. Green triangles represent mutation sites.

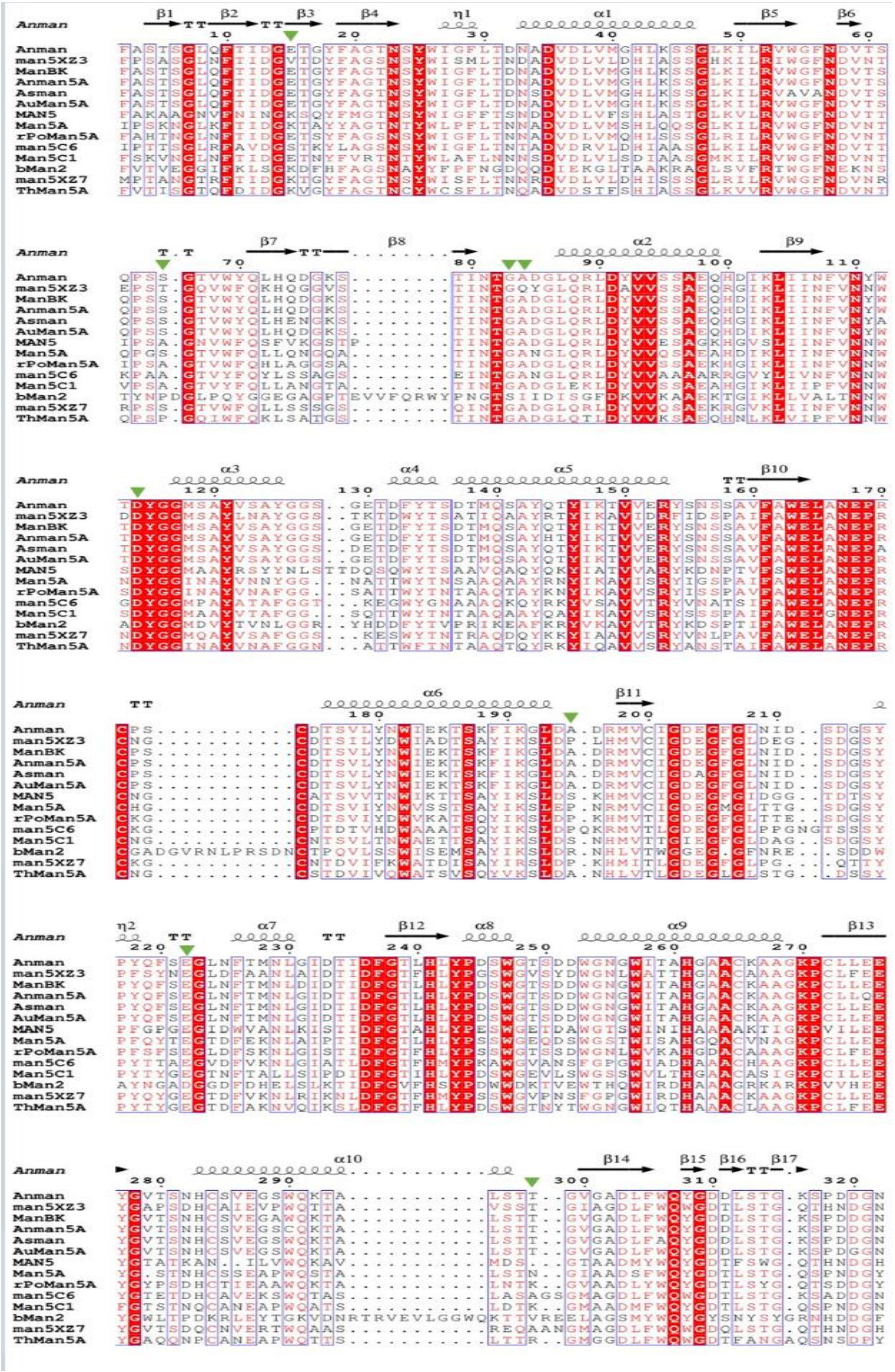

Figure S6, Kinetic fitting curves of Anman and Mut5.

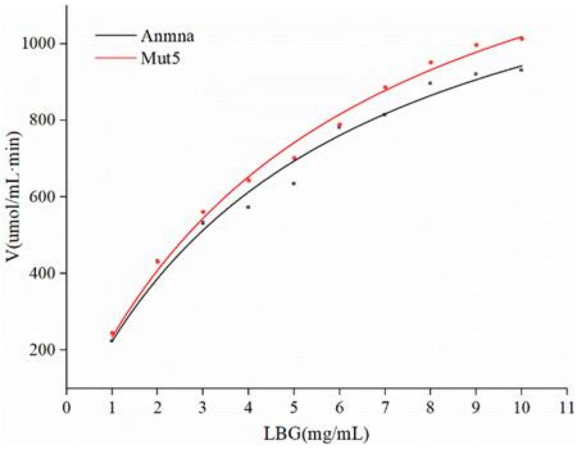

Figure S7, Energy changes of Anman and mutants in DSC.

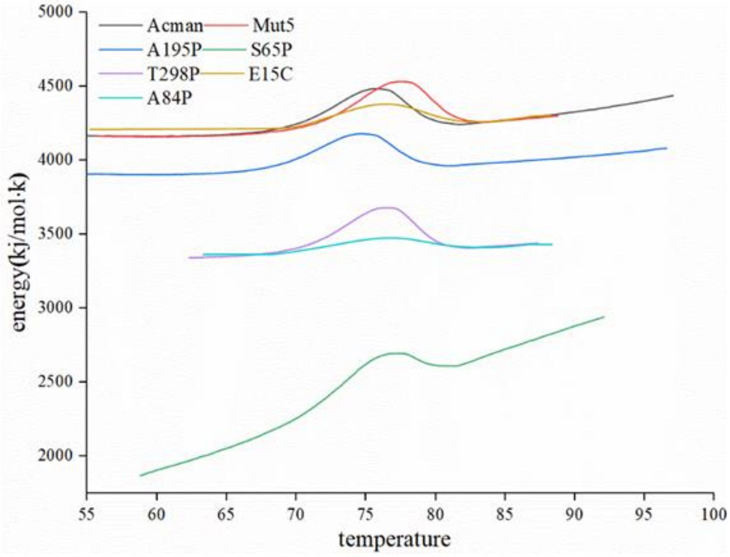

Figure S8, residual activity of Anman and Mut5 incubation at 75°C and 80°C for 10 min.

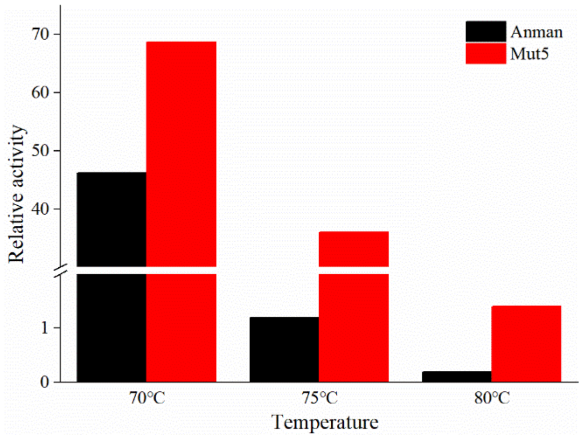

Figure S9, RMSD of Anman and Mut5.

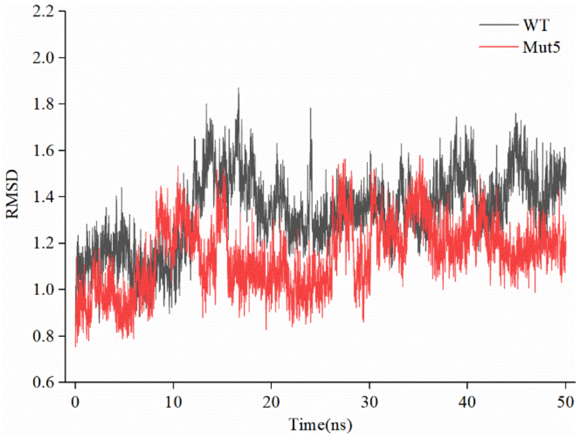

Supplement: Supplementary file 1 [file Data_Sheet_1.PDF]
